# Biorefinery of sunflower by-products: Optimization of twin-screw extrusion for novel biostimulants

**DOI:** 10.1016/j.heliyon.2025.e42576

**Published:** 2025-02-08

**Authors:** Jing Li, Hoang Khai Trinh, Lucas Tricoulet, Stéphane Ballas, Laurent Labonne, Danny Geelen, Philippe Evon

**Affiliations:** a HortiCell, Department Plants and Crops, Faculty of Bioscience Engineering, Ghent University, Coupure Links 653, 9000, Ghent, Belgium; b Laboratoire de Chimie Agro-industrielle, Université de Toulouse, INRAE, ENSIACET, 31030, Toulouse, France; c Ovalie Innovation, 2 Rue Marguerite Duras, 32000, Auch, France; d Faculty of Medicine, Nam Can Tho University, Can Tho City, Viet Nam

**Keywords:** Biorefinery, Biostimulant, Optimization, Plant bioassay, Sunflower, Twin-screw extrusion (TSE)

## Abstract

In view of improving the circularity and sustainability of crop production, sunflower by-products were extracted using twin-screw extrusion (TSE) to produce sunflower extract, a plant biostimulant that alleviates plant shoot development under salt stress conditions. The TSE process is a thermo-mechano-chemical pre-treatment method for the separation of liquid fraction from the biofiber. To improve the cost-efficiency of extraction, we determined the key procedure of TSE extraction within the production chain for biostimulants derived from sunflower bark and heads. This study scrutinizes sample preparation and extraction methods optimizing the sunflower by-product biorefinery, reducing energy input and maximal recovery of biostimulant activity. Optimal extraction conditions were obtained with starting material ground to a coarse size of 6 mm on average in alkaline aqueous solvent (pH 10) at a liquid-to-solid ratio of 5.5 injected at two different points using a 3 D length of reversed screw elements at the rotation speed of 200 rpm. These TSE settings provide a reproducible protocol for the biostimulant extraction from sunflower by-products. The optimized method contributes to improving the profitability of sunflower production and contributes to a more robust biostimulant extraction procedure.

## Highlights

• Sunflower bioconversion by TSE is validated to produce biostimulants.
• Plant bioassay-guided biorefinery is applied to optimize biostimulant production.
• Feedstock selection is based on the TSE extraction yield.
• TSE is optimized based on five key parameters.
• The technical assessment is made to define the best cost-profit balance.

## Graphical abstract

**Figure:**
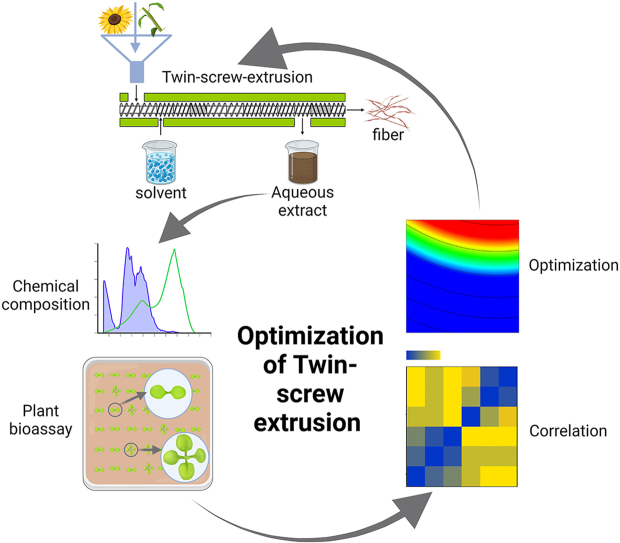

## 1 Introduction

Growing demand for human food, animal feed, and industrial crops causes an increase in productivity and diversification of agricultural waste with devastating impacts on the natural environment [1]. Crop residues generated during harvest and post-harvest account for approximately 30 % of total waste [2]. Sustainable agriculture advocates reintroducing plant biomass into the value-added production chain by means of clean biomass conversion approaches [3]. In the context of carbon neutrality, state-of-the-art biowaste biorefinery is a promising alternative for manufacturing fuels, materials, and chemicals derived from renewable resources [4]. The bioactive substances recovered from crop waste are being used in pharmaceuticals, cosmetics, food, industrial chemicals, and agricultural biologicals (*e.g.*, biostimulants and biopesticides) [[5], [6], [7]].

As denoted by the European Biostimulants Industry Council, biostimulants offer bioactive substances of natural origin that can improve plant growth performance and stress tolerance [8]. Experimental evidence and field trials typically show that biostimulants generally outperform in suboptimal growth conditions [9]. Plant extract-derived biostimulants take the lead in generating the best performance in enhancing crop biomass as determined by two meta-analyses [10,11]. Non-food plant tissues, also known as crop by-products and residues, are cost-effective options for raw materials to produce biostimulants, which is consistent with the circular bioeconomy concept [7].

Sunflower heads and stalks are historically used as alternative livestock roughage or organic mulch [12,13]. However, in today's practices, most of this material is left on the field after harvest. Natural fibers from sunflower stems have been upcycled for biocomposites, such as fiberboard [14,15]. Enzymatic protein hydrolysate from sunflower seed meal was shown to promote maize root development by a combination of bioactive compounds, including amino acids, humic acids, minerals, and sugars [16]. In previous research, we reported that sunflower bark extract (SBE), which is a side stream apart from fiber separation, is a promising biostimulant that promotes shoot growth and delays leaf senescence in salt-stressed Arabidopsis via true leaf assay [17]. Bark separation, as an additional step to isolate it from the inner pith in the sunflower stalk and made possible by the difference in density between these two solid fractions, introduces supplementary effort and cost. The protective effect of SBE might be attributed to its *in vitro* antioxidative activity scavenging free radicals. The presence of natural antioxidants, like phenolics and flavonoids, was detected in sunflower seed, floret, and leaf material [[18], [19], [20]]. Phytochemicals with antioxidant properties could aid crops in regulating cellular redox homeostasis mitigating abiotic challenges [21]. However, it is noteworthy that the concentrations of these antioxidants vary across different tissues. The biostimulant activity in other varieties of sunflower by-products remains unclear, posing a constraint on the cost-efficient selection of biomass feedstocks for further scale-up production.

Green extraction for bio-based chemicals is strongly mandated by the current regulations, followed by technological assessment of optimal raw material and energy consumption [22] When incorporating expected yields, profitability, and operating reliability during biorefinery modeling, the poor return-on-investment performance results in relatively few commercial biowaste conversion platforms [23]. Extrusion, for instance, is a commonly used thermo-mechanical biorefining technique for transforming biowaste into valuable components [[24], [25], [26]]. In terms of conditioning uniformity, energy efficiency, operational versatility (*e.g.*, addition of several liquids and/or solids at different locations along the extruder barrel), and cost-effectiveness, twin-screw extrusion (TSE) is generally more advantageous than single-screw extruders [[25], [26], [27], [28], [29]]. Lignocellulosic crop by-products are prominently utilized in the one-step TSE operation to produce biofiber [30]. And, when water injected is sufficient to allow the simultaneous collection of an aqueous extract, the latter is subsequently employed for the recovery of biochemicals [25]. While previous TSE operations have proven suitable for SBE extraction, it is essential to note that certain parameters necessitate optimization when producing other bioactive sunflower extracts. This optimization involves adjusting equipment settings and extraction conditions to achieve the desired outcomes of bioactivity, yield, and energy conservation. The reduced particle size of fine feedstock and increased liquid-to-solid ratio improve the efficiency of solvent penetration and solute diffusion [31]. Pretreatment with a mild alkaline solution is a sustainable approach for the biochemical extraction of compounds from lignocellulosic biomass. This method facilitates the breakdown of intermolecular ester bonds between lignin and hemicelluloses, enhancing the release of valuable constituents [32]. The screw profile encompasses the conveying, mixing, and mechanical shearing zones equipped with water injection point(s). The extracting zone intimates mixing between the liquid and the solid, and the pressing zone allows the liquid and solid phases to be continuously separated from each other. Rotation speed in the TSE design plays a critical role in determining the characteristics of the extrudate and influencing energy consumption [33]. Further optimization of the TSE process is imperative to achieve efficient biostimulant production, considering the critical operational conditions mentioned earlier. Most importantly, the rapid and robust plant assay serves as a pivotal tool for bioactivity screening, guiding the TSE operation cycle to produce biostimulants.

Here, we present the optimization of TSE settings for producing sunflower-derived biostimulants. The optimized procedures include sample preparation and selection, and extraction methods that enable reduced energy consumption and maximized recovery of biostimulant activity. More precisely, the influence of the sunflower tissue type will be first studied at constant TSE operating conditions. Then, using the most promising sunflower raw material, the TSE operating conditions will be optimized. The utilization of plant bioassay-guided TSE optimization, coupled with a trade-off in technical assessments, enables cost-effective optimization of feedstock properties and operational settings for biostimulant production.

## 2 Materials and methods

### 2.1 Raw material collection

A combine harvester adapted to sunflower (*Helianthus annuus*) was used to collect the heads and stalks and separate them concurrently into seeds and leftover biomass [34]. Stalks and seedless heads, representing around 33 % (w/w) and 15 % (w/w), respectively, of the dry aerial part of the sunflower plant, constituted the leftover biomass at the combine harvester outlet. In the fall of 2020 and 2021, they were collected together from Gers department, near Toulouse, southwest France, using a dedicated trailer directly attached to the combine harvester. By using this modified combine harvester completed with dedicated peripheral equipment allowing simultaneous separation of seeds on one side, and stalks and heads on the other, the total biomass yield per hectare was substantially increased from 600 to 900 kg of cultivation by-products (stalks and heads) in addition to 15 to 20 quintals of seeds depending on the plot. As a second step, they were then continuously separated from each other through a screening operation based on morphology to generate two additional raw resources, *i.e.*, stalks alone and heads alone. Raw materials were stored in ventilated bottom boxes until 10.7 % moisture content. No forced drying was applied to make the cost negligible before transferring to extrusion. Before extrusion, the raw materials were grinded using an Electra (France) Goulu N hammer mill fitted with grids made of circular holes of 6 mm or 20 mm opening diameter, resulting in two different levels for feedstock coarseness.

### 2.2 Optimization of feedstock and twin-screw extrusion operators

**Fig. 1:**
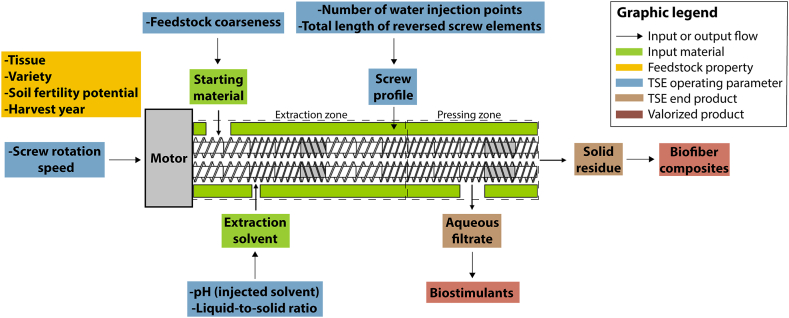
A biorefinery flowchart of sunflower by-products for biostimulants through twin-screw extrusion (TSE).

To identify robust starting material, raw resources varying in sunflower variety (linoleic (L) or oleic (O)) and tissue type (heads (H), stalks (S), bark (B), or heads plus stalks (HS)) were processed and followed by SBE18 described with technical details in Ref. [17] (Fig. 1, Table 1). Specifically, the oleic variety was cultivated in two adjacent fields with differing soil fertility potentials over two consecutive growing seasons (2020 and 2021). The same authors produced the SBE18 extract in stabilized conditions, meaning that the extrusion conditions (*i.e.*, screw and temperature profiles, inlet flow rates of sunflower bark and water, and screw rotation speed) were not changed [17]. In the present study, when evaluating the influence of sunflower tissue, the same methodology was adopted for identifying the most robust starting material.

**Table 1:** Sunflower extracts as feedstock from various tissue types, cultivation, and extraction solvents.

NO	Extract code	Starting material	Sunflower variety	Soil fertility potential	Harvest year	Extraction solvent^a^
1	SBE18^b^	Bark	Linoleic	High	2018	Water
2	BL20	Bark	Linoleic	High	2020	Water
3	BL20∗	Bark	Linoleic	High	2020	30 % (w/w) Ethanol
4	SL20	Stalks	Linoleic	High	2020	Water
5	HL20	Heads	Linoleic	High	2020	Water
6	HSL20	Heads + Stalks	Linoleic	High	2020	Water
7	HSO20	Heads + Stalks	Oleic	Low	2020	Water
8	HSO+20 (O1)	Heads + Stalks	Oleic	High	2020	Water
9	HSO+21(O15)	Heads + Stalks	Oleic	High	2021	Water

a The extraction solvent was injected continuously into the twin-screw extruder using a piston pump and then mixed intimately with the starting material.

b SBE18 originated from the same SBE stock as reported by Ref. [17].

The TSE-processed extracts were obtained using an industrial-capacity Clextral (France) Evolum HT 53 twin-screw extruder made of co-rotating and intermeshing screws (with a 53 mm screw diameter and a total barrel length of 36 times the screw diameter). The twin-screw extrusion treatment was performed under the following conditions: an inlet flow rate of sunflower tissues at 10 kg/h, a screw rotation speed of 250 rpm, screw profile 1 (Fig. S1), a 100 °C temperature in the extracting zone (modules 4 to 6), and a 110 °C temperature in the pressing one (module 8). Seven extracts from the 2020 sunflower harvest were processed using either aqueous or hydroalcoholic (30 % w/w ethanol) solutions. All liquid extracts were collected at the endpoint of TSE processing, followed by centrifugation, concentration via partial water evaporation, and finally freeze-drying to produce powder products for chemical composition and biostimulant activity assessment. Centrifugation was conducted using a centrifugal dryer (RC50PXR, Rousselet-Robatel, France) equipped with a 10 μm mesh bag at a rotation speed of 2000 rpm, with liquid extracts processed at a flow rate of approximately 15 kg/h. Concentration was achieved using a 300 L pilot unit (K9955 Tournaire, France), with partial water evaporation carried out under vacuum (80–100 mbar) and heated in a bain-marie to approximately 35 °C. The water evaporation rate was around 20 L/h, and the mass reduction of the clarified extract post-concentration was approximately 3–3.5 times. Finally, freeze-drying was performed using a 38 L pilot freeze-dryer (Cryonext, France), with the freezing temperature set at −30 °C and a vacuum applied at 0.3 mbar.

As the maximum extraction yield was obtained from HSO+20(O1) (Table S3), the TSE procedure was further customized from the same feedstock, this time by modulating five essential operating conditions in twin-screw extrusion processes (Table 2), while maintaining constant the inlet flow rate of raw material (10 kg/h), and the temperatures in the extracting and pressing zones (*i.e.*, 100 °C and 110 °C, respectively). Based on the past experience of TSE sunflower [[25], [26],^35^], the essential operating conditions are decided as feedstock coarseness, liquid-to-solid ratio, pH of the injected solution, screw rotation speed, as well as screw profile adapted for water injection points, and total length of reversed screw elements with the objective here of optimizing the liquid/solid separation efficiency (Fig. 1, Fig. S1). Therefore, the total length of reversed screw elements in the pressing zone was increased from 0.5 D to 3 D, to gradually increase the compression action on the liquid-to-solid mixture. A total of fourteen trials extracted from different parameter combinations (HSO+20(O1) to O14) were generated (Table 3). In addition, the optimal TSE operation (*i.e.*, O14) was repeated to produce O14b from the same feedstock of HSO+20, and HSO+21(O15) from the 2021-harvested batch to validate the biorefinery robustness.

**Table 2:** Operating parameters of the twin-screw extruder used for TSE optimization in biostimulant production.

NO.	TSE operating parameter		Unit	Level
1	Feedstock coarseness		mm	6, 20
2	Liquid-to-solid ratio		–	2.5, 4, 5.5
3	pH (injected solvent)		–	7, 10, 12
4	Screw profile	a. Number of water injection points (extraction zone)	–	1, 2
		b. Total length of the reversed screw elements (pressing zone)	D^a^	0.5, 1, 1.5, 3
5	Screw rotation speed		rpm	150, 200, 250

a Screw diameter (D) = 53 mm.

**Table 3:** TSE operating parameter settings for the optimization of twin-screw extrusion for HSO+20 extractions (trials HSO+20(trial O1) to trial O14b) and HSO+21 extraction (trial O15).

NO.	Extract trial code	Feedstock coarseness (mm)	Screw profile^a^	Number of water injection points	Total length of the reversed screw (D)	liquid-to-solid ratio	pH (injected solvent)	Screw rotation speed (rpm)
1	HSO+20 (O1)	20	1	1	0.5	4	7	250
2	O2	20	2	1	1	4	7	250
3	O3	20	3	1	1.5	4	7	250
4	O4	20	4	1	3	4	7	250
5	O5	20	5	1	1.5	4	7	250
6	O6	20	3	1	1.5	4	7	200
7	O7	20	3	1	1.5	4	7	150
8	O8	20	3	1	1.5	4	10	200
9	O9	20	3	1	1.5	4	12	200
10	O10	20	3	1	1.5	2.5	7	200
11	O11	20	3	1	1.5	5.5	7	200
12	O12	6	3	1	1.5	4	7	200
13	O13	20	6	2	1.5	4	7	200
14	O14	6	7	2	3	5.5	10	200
15	O14b^b^	6	7	2	3	5.5	10	200
16	HSO+21 (O15)^c^	6	7	2	3	5.5	10	200

a Screw profiles number 1 to 7 are described exhaustively in Fig. S1.

b Trial O14b is considered a technical repeat of trial O14.^c^ HSO+21 (trial O15) is considered a biological repeat of trial O14.

More importantly, the ultimate economic value of sunflower-derived biostimulants relies on the production yield and operating costs in addition to the investment in raw materials. The extraction yield in soluble molecules inside the clarified filtrate per kilogram of feedstock (R_MS_ (%)) and specific mechanical energy (SME (W h/kg feedstock)), were computed for each TSE operation according to Equation (1) and Equation (2). In continuous liquid-to-solid extraction through twin-screw extrusion, SME is considered an energy-sufficient indication since the major operating cost of TSE is composed of mechanical consumption [35].

Equation (1) $$Extraction\;yield\;\left(R_{MS}\right)=\left(Q_{F}\times T_{CF}\times {MS}_{CF}\right)/\left(Q_{s}\times \left(100-H_{s}\right)\right)$$

Where *Q*_*F*_ is the outlet flow rate of the filtrate (kg/h), *T*_*CF*_ is the content of the clarified filtrate in the filtrate (%), *MS*_*CF*_ is the dry matter content of the clarified filtrate (%), *Q*_*S*_ is the inlet flow rate of the starting material, and *H*_*S*_ is the moisture content of the starting material (%).

Equation (2) $$Specific\;mechanical\;energy\;\left(SME\right)=\left(U\times I\times \cos\;\varphi \times S_{S}\right)/\left(Q_{s}\times S_{\max}\right)$$

Where *U* is the operating voltage of the motor (*U* = 454 V), *I* is the current consumed by the motor (A), cos *φ* is the theoretical efficiency of the motor (cos **φ** = 0.9), *S*_*S*_ is the operating screw rotation speed (rpm), and *S*_max_ is the maximum screw rotation speed (*S*_max_ = 800 rpm).

Technical assessment of biorefinery was quantitatively evaluated on sunflower conversion into lyophilized powder, which could be valorized as biostimulants based on the optimal TSE operation. The remaining steps were regarded as conventional practices to assure robustness, such as milling for feedstock preparation before TSE, as well as afterward centrifugation, concentration, and lastly freeze-drying for the stabilization of active extracts. The material flow analysis took into account the input of biomass, energy, (alkaline) aqueous solution consumption, and the output of the end product (lyophilized powder) and other residues (adapted from Ref. [35]).

### 2.3 Chemical composition analysis and *in vitro* antioxidant activity determination

To ensure the quality of sunflower starting materials for TSE, various components were measured: moisture and dry matter content [37], mineral content [38], lipid content [39], and cell wall polymers (cellulose, hemicelluloses, and lignins) following van Soest and Wine [[40], [41]]. Additionally, spectrometric analysis was conducted on sunflower extracts to determine soluble proteins, digestible carbohydrates, total phenolics (expressed as Ascorbic acid (AsA) and Chlorogenic acid (CHA) equivalents), and total flavonoids (expressed as Quercetin and Rutin equivalents) as outlined in Ref. [17]. Despite the absence of a universally standardized method for measuring total antioxidant capacity in complex biological samples, the 2,2′-azino-bis (3-ethylbenzothiazoline-6-sulfonic acid) (ABTS) spectroscopic assay was used, as it is recommended for assessing both hydrophilic and lipophilic antioxidants [42]. The total *in vitro* antioxidant capacity was quantified using the IC50 (half-maximal inhibitory concentration) value equivalent to Trolox, and expressed as TEAC (Trolox equivalent antioxidant capacity) via the ABTS assay [43].

### 2.4 Arabidopsis true leaf assay

Biostimulant activity was assessed following the methodology described in Ref. [17]. Fifty Arabidopsis seeds were sown per plate, with three replicate plates for each treatment. The biostimulant efficacy was measured by evaluating Germination (%), True leaf formation (%), and Shoot area (SA in mm^2^). Sunflower bark extract from 2018 (SBE18) served as the positive control. To ensure the robustness of this plant assay, batch effects were assessed, given that SBE18 was tested across multiple assay batches. This bioassay was also employed to compare the biostimulant efficacy of three known antioxidants and three commercial biostimulants. The concentrations for the exogenous application of plant-origin antioxidants were selected based on their effects on stressed plants: 200 μM Glutathione (GSH) [44], 100 mM Ascorbic acid (AsA) [45], and 100 μM Quercetin [46]. One of the commercial biostimulants tested was Kelpak® (0.05 % v/v), a seaweed extract known for its beneficial effects on crop yield and quality [47,48]. The other two commercial biostimulants, 4-Good and 4-Terra, were tested at three different concentrations (1 %, 0.1 %, and 0.01 % v/v) and are registered under EU regulations [49] (additional details in Table S1).

### 2.5 Statistical analysis

Variables were initially assessed for normal distribution and homogeneity. One-way ANOVA analysis with post hoc Tukey HSD test (*α* = 0.05) was used for parametric comparisons. To accommodate for non-linear relationships between multiple variables, Spearman's correlation was applied to measure the underlying trends [50] via GraphPad Prism version 9.0.0 (San Diego, USA). For each pair of parameters, the strength of correlation was represented by the coefficient *r*_*s*_, and the *p*-value indicated the statistical significance of the correlation probability at a 95 % confidence interval. The Heatmap matrix was illustrated at range scale [−1, 1] (blue to orange) of *r*_*s*_. For TSE optimization, the customized design with response surface methodology in Design-Expert version 12 software was utilized [51]. Five TSE factors of varying levels in Table 2 were analyzed by applying linear regression models with R_MS_ (%), SME (W h/kg), and representative biostimulant activities (True leaf (%) and SA (mm^2^)) as responses. All the models are significant (*p* < 0.05) in ANOVA (analysis of variance) tests and the plots were then produced.

## 3 Results and discussion

### 3.1 Robustness of the rapid plant bioassay

The objective of this study was to optimize sunflower feedstock and TSE operating conditions, using plant bioassays to maximize biostimulant activity. The initial step involves validating the robustness of the plant assay employed to screen biostimulant activity. With consistent results observed across four independent runs of the same assay testing on SBE18, it can be asserted that the plant assay is proficient in screening similar bioactivity in other samples akin to SBE18 (Table S2). Subsequently, the sunflower-derived extracts were screened and evaluated for biostimulant activity, and extracted first from different plant tissues and then under different TSE settings, guided by the target bioactivities gained from the validated plant assay.

### 3.2 Feedstock selection and the consistency of sunflower biomass

**Fig. 2:**
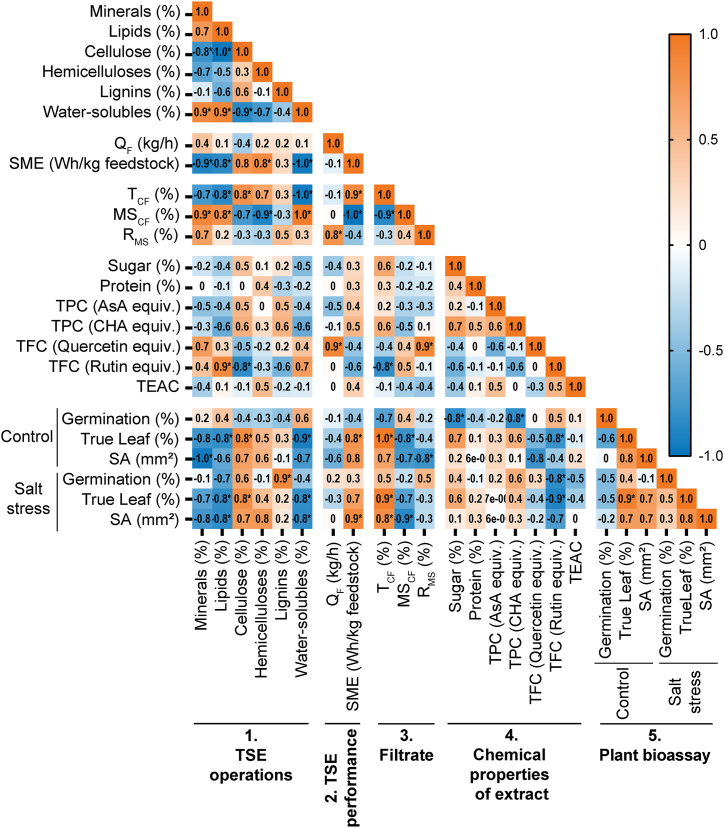
Heatmap of Spearman's correlation coefficient of seven sunflower extracts derived from different tissues harvested in 2020 **(**mentioned in Table 1).

The availability of biomass and sustainable supply chains of crop residue are crucial factors determining the raw material cost of agricultural biorefinery manufacturing [52]. To determine the consistency of sunflower by-products, seven different feedstocks were tested, varying the sunflower variety (linoleic or oleic), biomass type (bark, stalks, or heads plus stalks), soil fertility potential (high or low), and harvest year (2018, 2020 or 2021) (Table 1). Five TSE-produced extracts were obtained from standard but non-optimized TSE operating conditions allowing stable extrusion process over time, *i.e.*, an inlet flow rate of sunflower tissues of 10 kg/h, a screw rotation speed of 250 rpm, screw profile 1, and temperatures in the extracting and pressing zones of 100 °C and 110 °C, respectively. They were then compared on the basis of their chemical characteristics, TSE performance, filtrate treatment, chemical properties of extract, and plant bioassay of biostimulant efficacy (Fig. 2).

The comparison is illustrated using a heatmap matrix of Spearman's correlation coefficient, which displays the pair correlation of all variables (Fig. 2). The R_MS_ positively correlated to minerals, lipids, and water-solubles, but negatively correlated with cellulose and hemicelluloses. The highest extraction yield was reached from the heads plus stalks biomass source of oleic sunflower (Table S3). The combination of fibrous particles of varying lengths (longer ones from the stalks, and shorter ones from the heads) visibly improves the efficiency of liquid-to-solid separation at the level of the CF2C reversed pitch screws, as previously observed when extracting vegetable oil and other biopolymers (*e.g.*, proteins, and pectins) from whole plant using water [35]. Sunflower biomass has been shown to be rich in cellulose and lignin and poor in hemicelluloses when grown in highly fertile, nitrogen-rich soil. Whole stalks and barks contain lower water-soluble content whereas a larger amount of cellulose and hemicelluloses, which are the two main whole-crop and woody biorefinery products, together with other value-added biochemicals [53]. Feedstocks of sunflower bark or stalk with higher fiber content as a sum of cellulose, hemicelluloses, and lignin increase the motor's energy demand, resulting in a higher SME (up to 603 W h/kg starting material) but in a lower MS_CF_ (2.7 % max). It is noted that extracts resulting in higher bioactivity contain a higher portion of bark composite (BL20 and BL20∗) with cellulose and hemicelluloses. The reason might be an increase of the digestible carbohydrates, soluble proteins, and phenolics while a decrease in flavonoids and *in vitro* antioxidant content (TEAC) were detected in bark extract that aligned with the biostimulant activity of True leaf and SA. However, the isolation of bark from the stalk requires its pre-treatment through grinding, de-dusting, and then aspiration of pith particles, resulting in extra operating cost [17]. Therefore, the study was continued with HSO+20 from whole stalks plus heads as the cost-minimizing feedstock because of its optimal yield per unit of biomass at an acceptable expense of bioactivity. In other words, with HSO+20, more sunflower feedstock can be collected in the field, without the additional cost of separating bark from pith and heads before TSE. In addition, the R_MS_ extraction yield is high (11.9 %), and the biostimulant activity of SA of the corresponding extract is effective under salt stress conditions (Table S3).

The color in each cell indicates the coefficient *r*_*s*_. Parameters in plant bioassay are represented as biostimulant efficacy. The asterisk (∗) indicates the significance of the correlation (*p* < 0.05 at 95 % confidence interval). TSE: Twin-screw extrusion; Q_F_: filtrate outlet flow rate; T_CF_: content of the clarified filtrate in the filtrate (%); MS_CF_: dry matter content of the clarified filtrate (%); R_MS_: extraction yield in soluble molecules inside the clarified filtrate per kilogram of feedstock; TPC: total phenolic content; AsA: ascorbic acid; CHA: chlorogenic acid; equiv.: equivalent; TEAC: Trolox equivalent antioxidant capacity; SME: specific mechanical energy; SA: shoot area.

### 3.3 Twin-screw extrusion optimization and consistency

From the previously selected optimal sunflower feedstock (*i.e.*, HSO+20), in a set of additional experiments, an optimization of the TSE operating conditions was then conducted. The impacts of feedstock coarseness (6 mm or 20 mm), liquid-to-solid ratio (from 2.5 to 5.5), pH of injected water (from 7 to 12), and screw rotation speed (from 150 to 250 rpm) were tested. Seven different screw profiles were also tested (1–7) and categorized according to the number of water injection points (1 or 2), and the total length of reversed screw elements (from 0.5 D to 3 D) (Table 3). The TSE performance and the biostimulant activity were then evaluated (Table S4).

Screw profile is critical to affecting TSE performance in this study. The extracting zone was made of one or two series of bilobe paddles associated with the number of water injection points after the feeding zone, favoring intimate mixing between the solvent and the solid biomass. For its part, the pressing zone situated at the end of the screw profile (module 8), immediately downstream of the filtration module (module 7), allows the continuous separation of the liquid and solid phases thanks to the implementation of one or more pair of conjugated reversed pitch screws. The type of reversed screw elements differs in their pitch and total length (Fig. S1), providing a variable pressure buildup [29]. The influence of the pressing zone was first evaluated through trials HSO+20(O1) to O5, always using one series of bilobe paddles, one water injection point, water (pH 7) as the injected solvent at a liquid-to-solid ratio of 4, and a screw rotation speed of 250 rpm. With the increasing length of CF2C reversed screw elements obtained by using more screw pairs, from trial HSO+20(O1) to O4, the dry matter content of the clarified filtrate (%) (MS_CF_) and SME tended to gradually increase, and they were both maximal with six pairs of CF2C screws (screw profile 4). However, this was not the case in extraction yield of soluble molecules (Table S4). Instead, the rest of the content of insoluble dry matter because of more intensive compression on the mix would contribute to biofiber production. The higher mechanical shear applied by the single-flight reversed screw elements (CF1C) used in trial O5 produced quite similar MS_CF_ and R_MS_ in comparison with trial O3 obtained from the same length of reversed screw elements (1.5 D) consisting of the double-flight screws (CF2C). However, trial O5 consumed much more energy than trial O3 (+34 % for SME, which is far from negligible). Meanwhile, all other operating conditions being equal (*i.e.*, 20 mm for feedstock coarseness, three pairs of CF2C screws in the pressing zone, 4 for liquid-to-solid ratio, 7 for pH of the injected solvent, and 200 rpm for screw rotation speed), screw configuration with two water injection points, each followed by a series of bilobe paddles (trial O13), benefits most of the evaluations instead of one injection point (trial O6) despite a comprise in energy expense (Table S4). Screw profile 7 was therefore decided as the more efficient option, combining two water injection points and two series of bilobe paddles in the extracting zone, and 3 D length of double-flight reversed screw elements (CF2C) in the pressing zone (Fig. S1).

When comparing trials O3, O6, and O7, all three using screw profile 3 and the same operating conditions with the exception of screw rotation speed, a decrease in the latter increased gradually the extraction efficiency and biostimulant activities while reducing the energy consumption (Table S4). In practice, a bit higher screw rotation speed of 200 rpm reduces the risk of machine clogging over time. Other operational parameters also matter. On the one hand, larger liquid-to-solid ratio positively affects the TSE performance based on trials O6, O10, and O11, *i.e.*, the more the liquid-to-solid ratio, the more the R_MS_ extraction yield, and the more the biostimulant activity of True leaf and SA under salt stress conditions. On the other hand, the most potent biostimulant activity found in trial O9 was extracted from a strong basic solvent (pH 12) in contrast to pH 10 used in O8 or to pH 7 used in O6.

**Fig. 3:**
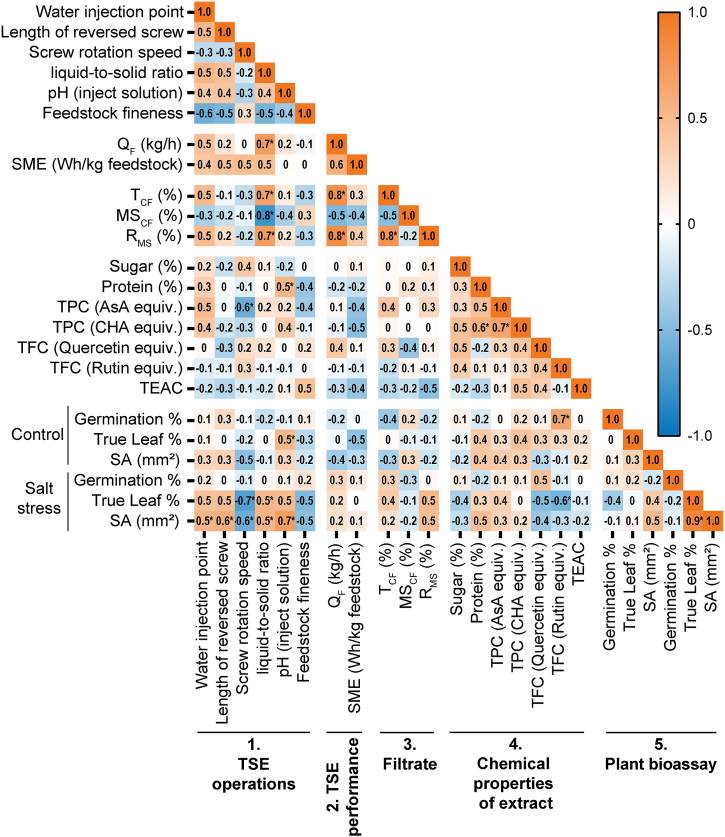
Heatmap of Spearman's correlation coefficient of sunflower extracts using fourteen different TSE operation conditions from HSO+20 (mentioned in Table 2).

Fig. 3 shows the Heatmap of Spearman's correlation coefficient using the fourteen different TSE operation conditions from HSO+20. With *r*_*s*_ > 0.5, R_MS_ positively correlated with the number of water injection points and liquid-to-solid ratio. It confirms the expected better diffusion of the solvent within the solid particles when it is injected gradually in two consecutive points, simultaneously with the higher extraction yield obtained when more solvent is injected. While the other correlations with other factors were weaker (0 < *r*_*s*_ < 0.5). The correlation of bioactivities trends with TSE operators was consistent with the extraction yield, where screw rotation speed and liquid-to-solid ratio play significant roles. Additionally, SME positively correlated with four operations regardless of the pH of the injected solution or the feedstock coarseness.

**Fig. 4:**
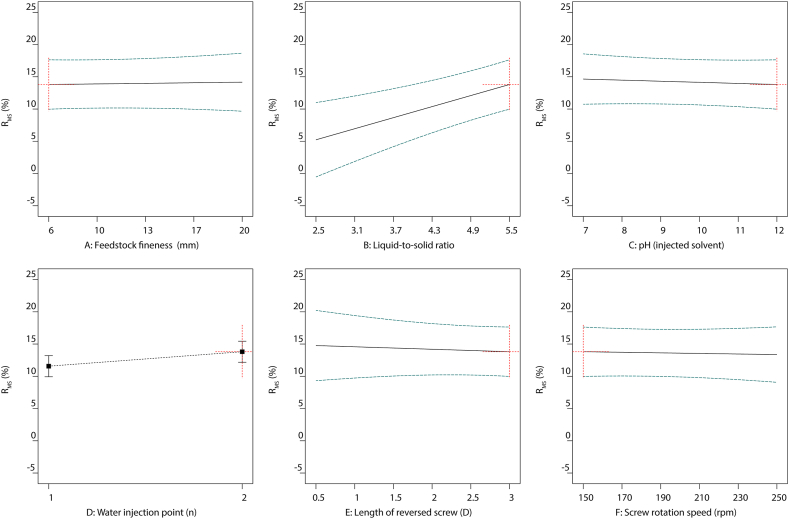
All factors plot of R_MS_ (%) under six different TSE operation conditions with the optimal settings of feedstock coarseness (20 mm), liquid-to-solid ratio (5.5), pH (injected solvent) (7), water injection point (2), length of reversed screw (0.5 D), and screw rotation speed (150 rpm). TSE: twin-screw extrusion; R_MS_: extraction yield in soluble molecules inside the clarified filtrate per kilogram of feedstock.

**Fig. 5:**
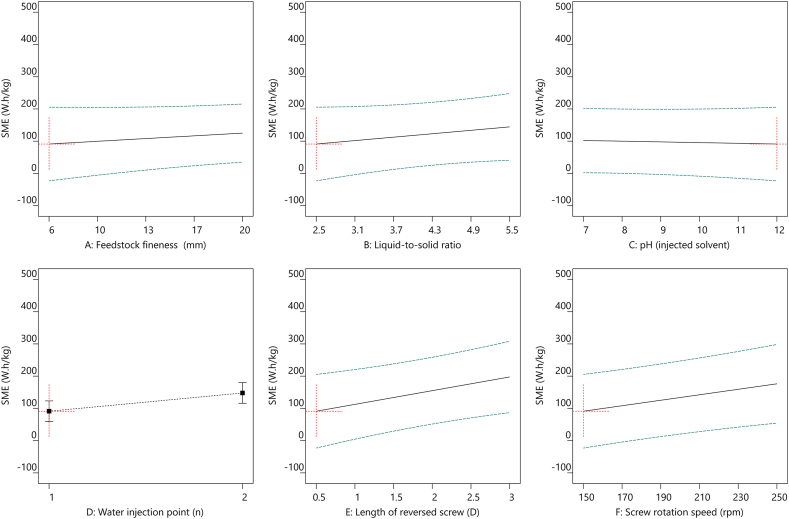
All factors plot of SME under six different TSE operation conditions with the optimal settings of feedstock coarseness (6 mm), liquid-to-solid ratio (2.5), pH (injected solvent) (12), water injection point (1), length of reversed screw (0.5 D), and screw rotation speed (150 rpm). TSE: twin-screw extrusion; SME: specific mechanical energy.

**Fig. 6:**
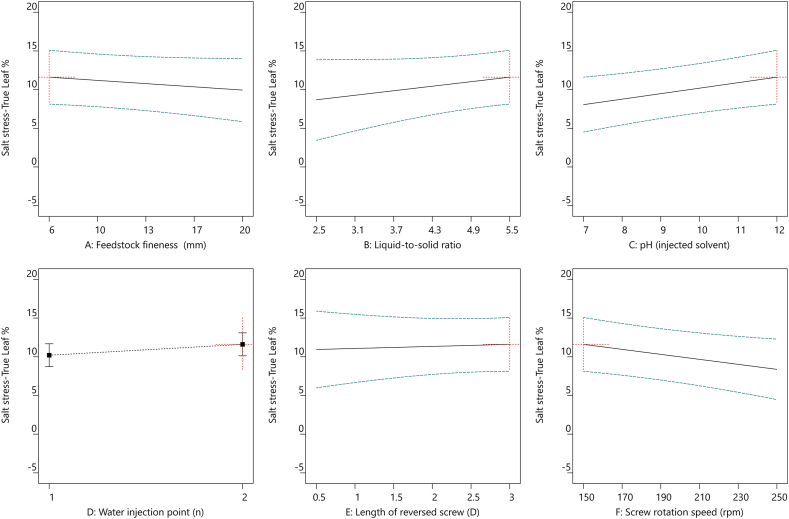
All factors plot of True leaf (%) under six different TSE operation conditions with the optimal settings of feedstock coarseness (6 mm), liquid-to-solid ratio (5.5), pH (injected solvent) (12), water injection point (2), length of reversed screw (3 D), and screw rotation speed (150 rpm). TSE: twin-screw extrusion; True leaf (%): percentage of plants with the first pair of true leaves.

**Fig. 7:**
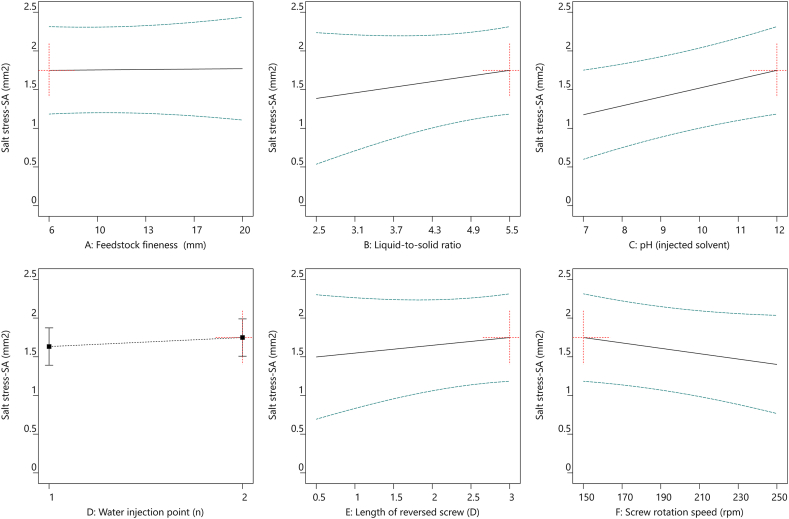
All factors plot of SA under six different TSE operation conditions with the optimal settings of feedstock coarseness (6 mm), liquid-to-solid ratio (5.5), pH (injected solvent) (12), water injection point (2), length of reversed screw (3 D), and screw rotation speed (150 rpm). TSE: twin-screw extrusion; SA: total shoot area.

In accordance with the extraction optimization of TSE operating parameters, multiple linear regression models were performed to maximize the utilization of the extraction procedure and minimize the energy consumption. This enabled to plot the evolutions of R_MS_ extraction yield (Fig. 4), SME (Fig. 5), True leaf (Fig. 6), and SA (Fig. 7) as functions of the six different TSE operation conditions, *i.e.*, feedstock coarseness (A) (Fig. 4, Fig. 5, Fig. 6, Fig. 7A), liquid-to-solid ratio (B) (Fig. 4, Fig. 5, Fig. 6, Fig. 7B), pH of the injected solvent (C) (Fig. 4, Fig. 5, Fig. 6, Fig. 7C), number of water injection points (D) (Fig. 4, Fig. 5, Fig. 6, Fig. 7D), length of reversed screws (E) (Fig. 4, Fig. 5, Fig. 6, Fig. 7E), and screw rotation speed (F) (Fig. 4, Fig. 5, Fig. 6, Fig. 7F). The optimized settings for R_MS_ (Fig. 4) are a liquid-to-solid ratio at 5.5 (Fig. 4B) with two water injection points (Fig. 4D), while the other parameters have a minor impact. In parallel, to minimize SME (Fig. 5), the TSE operations set as feedstock coarseness at 6 mm (Fig. 5A), liquid-to-solid ratio at 2.5 (Fig. 5B), one injection point (Figs. 5D), 0.5 D length of reversed screw elements (Fig. 5E), and screw rotation speed at 150 rpm (Fig. 5F). Conclusively, the optimal settings of TSE parameters required trade-off considerations with the aims of the highest extraction yield and lowest energy consumption.

More importantly, the biorefinery optimization required suitable unit operations to achieve the desired biostimulant activities in sunflower extracts. A similar pattern of correlations with TSE operations was observed in the two bioactivity responses, True leaf and SA, regardless of the control or salt-stressed conditions in the Arabidopsis true leaf assay (Fig. 3). The bioactivity of True leaf (Fig. 6) and SA (Fig. 7) under salt stress conditions was significantly increased under TSE conditions of liquid-to-solid ratio at 5.5 (Fig. 6, Fig. 7B), alkaline solvent (pH 12) (Fig. 6, Fig. 7C), and a slower screw rotation speed (150 rpm) (Fig. 6, Fig. 7F) with two injection points (Fig. 6, Fig. 7D). While the aimed bioactivities remained relatively stable in conditions as the finer feedstock and increased of the total length of the reversed screws.

Employing the linear models, the best compromise for efficient bioconversion processing of sunflower extracts can be identified, with the aim of high extraction yield (Fig. 4) and potent activities of salt-stressed True Leaf (Fig. 6) and SA (Fig. 7) consuming low energy (Fig. 5). Optimal operation conditions can be summarized as follows: 6 mm of feedstock coarseness (with the objective of lowered SME), liquid-to-solid ratio at 5.5 (with the objective of higher R_MS_ extraction yield), basic pH in a range of 10–12 (with the objective of increased bioactivities), two water injection points in the extracting zone (again with the objective of higher R_MS_ extraction yield), 3 D length for the reversed screws in the pressing zone (with the objective of maximizing the pressing effect of the CF2C reversed screws on the liquid-to-solid mixture), and 150 rpm for screw rotation speed (again with the objective of increased bioactivities while minimizing SME). Such optimal condition is, specifically, associated with a low screw rotation speed, which would limit the incoming flow of sunflower biomass to avoid clogging the machine over time. TSE productivity would therefore be limited in the outflow of active molecules.

Considering all the abovementioned aspects, the operation condition applied in the production of the O14 extract appears to be more robust for future industrial use as a slightly higher screw rotation speed was chosen (200 rpm instead of 150 rpm) to avoid the machine clogging over time (Table S4). The production trial O14 was extracted after 4 h of stable TSE operation without clogging the filtering grids. Moreover, trial O14 represented the highest extraction yield (13.6 %), simultaneously with the most promising bioactivities of True Leaf and SA under salt stress conditions. The TSE operational parameters of O14 were thusly considered as our optimized biorefinery of sunflower by-products for later technical assessment.

The color in each cell indicates the coefficient *r*_*s*_. Parameters in plant bioassay are represented as biostimulant efficacy. The asterisk (∗) indicates the significance of the correlation (*p* < 0.05 at 95 % confidence interval). TSE: Twin-screw extrusion; Q_F_: filtrate outlet flow rate; T_CF_: content of the clarified filtrate in the filtrate (%); MS_CF_: dry matter content of the clarified filtrate (%); R_MS_: extraction yield in soluble molecules inside the clarified filtrate per kilogram of feedstock; TPC: total phenolic content; AsA: ascorbic acid; CHA: chlorogenic acid; equiv.: equivalent; TEAC: Trolox equivalent antioxidant capacity; SME: specific mechanical energy; SA: shoot area.

Next, we evaluated the robustness of the technical TSE operation and the consistency of productivity and bioactivity in the feedstock. The O14 TSE optimal extraction setting was technically repeated from the same feedstock (HSO+20), and produced O14b extract (Table S5). In fact, really comparable results were obtained between the O14 and O14b trials for SME, R_MS_ extraction yield, chemical composition of the extracts, and their bioactivity under salt stress conditions. Trial O15 was obtained from the 2021 harvest feedstock batch (HSO+21) under the same TSE operations. The TSE performance of O15 generated quite similar results with the chemical profiles of the extracts, in line with their activities. It is however important to note that HSO+21(O15) resulted in a reduction in R_MS_ (9.2 % instead of 13.6 % for HSO+20), and consumed a higher SME value (516 W h/kg instead of 343 W h/kg). The reason is presumably that HSO+21 contained fewer hemicelluloses and water-solubles, leading to a reduction in extractable molecules. In parallel, the higher amount of lignocellulose caused higher pressure buildup in the pressing zone and, as a consequence, the increased energy consumption. These results reveal the importance of repetitive chemical and bioactivity analysis to evaluate reproducibility over different harvest years.

The use of annual crop biomass as feedstock has some drawbacks, such as seasonal production cycles and the stabilization requirements prior to storage [54]. Furthermore, handling and storage of biomass cost, variability, and risk in the supply chain and logistics must also be taken into account [55]. Biowaste management in flexible bio-based supply chain networks therefore requires improvement for continuous TSE production, which includes the securing of biomass production, collection, storage, and transportation [56]. The variability of raw material batches, depending on the geographical location of the plot and/or the year of harvest, will therefore have to be considered for future industrial developments. Standardization of raw material treatment provides an informed basis for downstream TSE optimization and further pilot-scale production.

For additional work, a full factorial design of experiments would be also needed to unlock the full potential of process optimization [57]. In particular, other TSE conditions such as processing temperature and feed rate, both chosen to be constant in the present study, are also factoring that impact on biomolecule recovery [58]. The maximal productivity of the industrial-capable TSE would demand further optimization to achieve a higher profit-to-cost ratio for the extrusion process [26,59].

### 3.4 Technical assessment of sunflower biorefinery

**Fig. 8:**
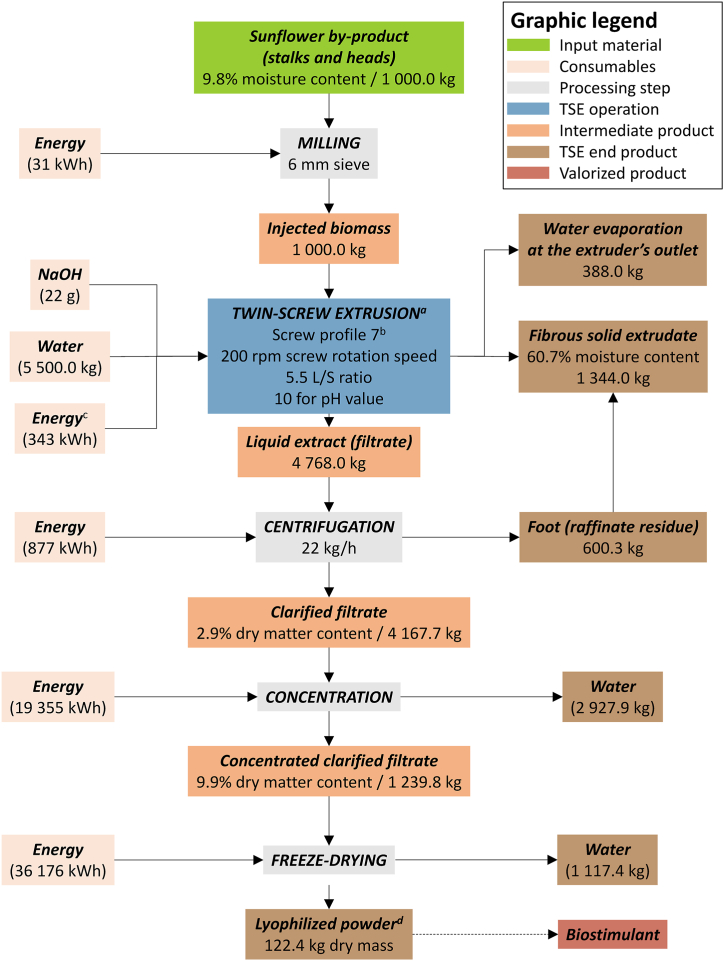
Material flow analysis and energy cost of sunflower conversion into lyophilized powder used as biostimulants. ^a^ The Twin-screw extrusion (TSE) operation conditions were of the O14 trial conducted from the HSO+20 sunflower feedstock (Table 3). ^b^ Screw profile 7 was described exhaustively in Fig. S1^c^ Energy consumption during TSE was estimated by means of specific mechanical energy (SME). ^d^ Lyophilized powder yield was calculated based on the extraction yield (R_MS_ (%)). TSE: twin-screw extrusion; R_MS_: extraction yield in soluble molecules inside the clarified filtrate per kilogram of feedstock.

The material flow analysis was applied to illustrate the optimized TSE extraction for biostimulant production derived from sunflower by-products. Utilizing stalks and heads aims to yield a larger quantity of starting biomass. Energy input calculations determine the operation cost including milling of sunflower feedstock, TSE operation and the downstream processes such as filtrate centrifugation, concentration of the clarified filtrate, and freeze-drying of the concentrated clarified filtrate. As discussed before, trial O14 from the HSO+20 feedstock was used as it corresponded to the maximal value for extraction yield and considerable bioactivity of the entire study (Fig. 8). The first three stages of the process consume much less energy that is negligible for grinding, 0.6 % for TSE, and 1.5 % for centrifugation, while concentration and freeze-drying are costly, representing 34 % and 64 %, respectively, of the total energy consumption of the process. Next, we explored the powder product of sunflower extract in liquid (solution and suspension), or solid formulations based on its physiochemical properties (*i.e.*, solubility, stability, viscosity, etc.). The use of the biostimulant liquid extract generated directly at the end of centrifugation (*i.e.*, the clarified filtrate) is preferred for cost-efficient industrial biorefinery, though the lyophilized product can conserve a longer shall life of active ingredients [60]. The left solid fraction is valorized into various bio-based materials such as fiberboards [61,62], thermal insulation, and thermoplastic (bio)composites [62]. An economic and environmental evaluation of the entire biorefinery process, including capital and operation costs, revenues, and life cycle analysis is further required for a final assessment [63].

In particular, the costs to consider when scaling up the technology to a higher TRL (Technology Readiness Levels) include.

(i) The raw material costs: sunflower by-product, NaOH, and water.
(ii) The production cost of the extract; here, if not freeze-dried, the production cost for the clarified filtrate is estimated at 1251 kWh for 4768 kg of clarified filtrate with 2.9 % dry matter content (Fig. 8), equating to 0.262 kWh/kg or 0.066 €/kg based on an electricity price of 0.25 €/kWh (average electricity price based on the energy mix in France in 2023).
(iii) The capital expenditure (CAPEX): costs associated with purchasing equipment such as the hammer mill, twin-screw extruder, centrifugal dryer, and optionally a concentrator for partial water evaporation or a freeze-dryer (or atomizer).
(iv) The operational expenditure (OPEX): primarily related to the twin-screw extrusion process, including replacement of wear-prone parts like screw elements, sleeve modules, and filter grids, which may degrade over time due to abrasion.

### 3.5 Comparison of biostimulant activity between sunflower extracts, known antioxidants, and commercial products

**Fig. 9:**
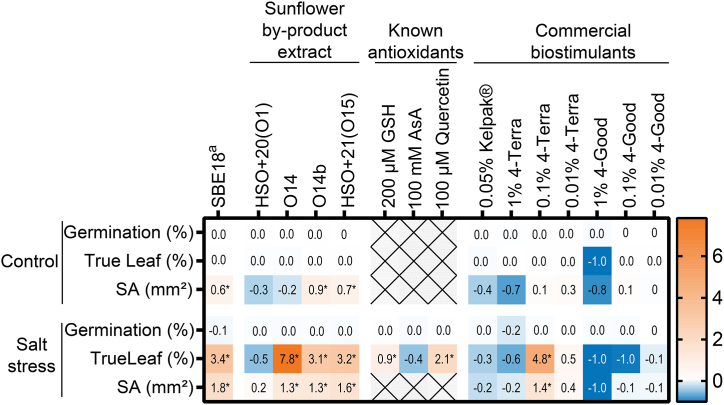
Heatmap matrix on the biostimulant efficacy of sunflower-derived extracts, known antioxidants, and commercial biostimulants. ^a^ SBE18 was referred to the bioassay batch 4 (Table S2). Asterisk (∗) indicates the statistical significance (*p* < 0.05 at 95 % confidence interval) compared to blank under control or salt stress conditions. ( × ) denotes missing data. The concentration of 0.5 g/L was used for both SBE18 and other sunflower extracts. The codes for sunflower extracts are shown in Table 1, Table 3. SA: shoot area; GSH: glutathione; AsA: ascorbic acid.

The extract SBE18 was used as the sunflower extract reference across different batches of bioassays to compare with known antioxidants and commercial biostimulants. The sunflower extracts, O14, O14b, and HSO+21(O15), show similar activities to SBE18 except HSO+20(O1) (Fig. 9). Plants were better protected from salt stress with 100 μM Quercetin than with 200 μM GSH, while no protective effect was observed in plants treated with 100 mM AsA. Quercetin likely exerts superoxide anion scavenging activity, reflecting a lower redox ratio and supporting the transition from cell proliferation to cell differentiation [64,65]. The commercial biostimulant 4-Terra at 0.1 % concentration showed bioactivity comparable to SBE18 but not 0.05 % Kelpak®. Both extracts contain organic acids and heterosides [17,66]. The U-shaped dose response of 4-Terra addresses the importance of the redox balance in regulating plant meristems for true leaf development [67]. The active sunflower extracts showed slightly higher biostimulant activities compared to 1 % of 4-Terra and 100 μM Quercetin.

Nevertheless, it is essential to note that the concentration and timing of biostimulant applications play a critical role in determining their biological performance [11]. Some commercial biostimulants offer recommended field doses tailored for specific groups of crop species, as illustrated in Table S1. The intricate composition of complex mixtures in biostimulants complicates the investigation of the mode of action of active ingredient(s) and the corresponding application methods. Conducting an in-depth dose-response-time study on the mechanism of biostimulant activity can yield more robust evidence, translating into effective functionality in the field.

For the following research, the *in vivo* antioxidant level is more accurate as a monitoring measure of stress resistance. Due to the limitations of membrane interaction with chemicals and cellular uptake efficiency, it is unrealistic to linearly predict the *in vivo* antioxidant activity in living organisms from the *in vitro* antioxidant capacity of the exogenous applications, although the tendency is generally positive at nontoxic doses [68,69]. The *planta* antioxidant activity was extensively applied in plant cells indicating the real-time redox state of living organisms by chemical probes or genetically encoded biosensors expressing redox-sensitive markers [70]. Next, the *in vivo* redox changes at a cellular or whole plant level under salt stress will be quantitatively evaluated in the presence of sunflower extracts. For instance, hyper 7 as a sensitive H_2_O_2_ biosensor could be used to monitor plant stress levels through fluorometric measurements via a hyperspectral camera. Regarding practical scenarios of biostimulant applications, the knowledge gained from the Arabidopsis true leaf assay should be further expanded to other crop species at different growth stages to cope with its function of enhancing stress tolerance.

## 4 Conclusions

In anticipation of the growing problem of non-renewable resource shortage and concerns regarding environmental impact, crop by-products are considered a source of biomolecule alternatives. The extraction of these molecules depends on technically challenging biorefinery methods. We evaluated TSE as a method to extract biomolecules from sunflower cultivation by-products of heads and stalks. The treatment of sunflower biomass through TSE resulted in the optimized production of biostimulants at reasonable production cost of 0.26 kWh/kg of clarified filtrate with 2.9 % dry matter content. The suggested procedure starts from small coarse particles (6 mm), injects alkaline solution (pH 10) to achieve 5.5 liquid-to-solid ratio at two points, and operates a 3 D length of reversed screws at 200 rpm screw rotation speed. This study is the first assessment of the efficiency and energy cost associated with upscalable TSE extraction of bioactive molecules from sunflower by-products. The reduced complexity associated with maintaining consistent feedstock, optimizing TSE operations, and deciphering the composition of active compounds removes a bottleneck barrier to the further development of novel biostimulants derived from sunflower by-products.

## CRediT authorship contribution statement

**Jing Li:** Writing – original draft, Visualization, Validation, Methodology, Investigation, Formal analysis, Data curation. **Hoang Khai Trinh:** Investigation. **Lucas Tricoulet:** Resources, Methodology, Investigation. **Stéphane Ballas:** Resources, Methodology, Funding acquisition. **Laurent Labonne:** Methodology, Investigation, Formal analysis, Data curation. **Danny Geelen:** Writing – review & editing, Supervision, Project administration, Funding acquisition, Conceptualization. **Philippe Evon:** Writing – review & editing, Project administration, Methodology, Investigation, Funding acquisition, Formal analysis, Data curation, Conceptualization.

## Data availability statement

Data included in the article/supplementary material is referenced in the article.

## Declaration of competing interest

The authors declare the following financial interests/personal relationships which may be considered as potential competing interests:Danny Geelen and Philippe Evon report on financial support provided by the FACCE-SURPLUS European Program. Danny Geelen reports financial support provided by the 10.13039/100012331Flanders Innovation and Entrepreneurship in Belgium. Philippe Evon reports financial support provided by 10.13039/501100001665Agence Nationale de la Recherche in France. Jing Li reports a relationship with 10.13039/501100004543China Scholarship Council (China), and Special Research Fund of 10.13039/501100004385Ghent University (Belgium) that include funding grants. Danny Geelen and Philippe Evon are co-inventors of the patent “Sunflower bark extract and uses thereof”, licensed to US 2022/0408733 A1.
